# Studies on Square Wave and Cyclic Voltammetric Behavior of 1,2- and 1,4-Dihydroxybenzenes and Their Derivatives in Acetic Acid, Ethyl Acetate and Mixtures of the Two

**DOI:** 10.3390/mps7060102

**Published:** 2024-12-20

**Authors:** László Kiss

**Affiliations:** 1 Department of Organic and Medicinal Chemistry, Faculty of Pharmacy, University of Pécs, Honvéd Street 1, H-7624 Pécs, Hungary; kissl@gamma.ttk.pte.hu; 2 János Szentágothai Research Center, University of Pécs, Ifjúság Street 20, H-7624 Pécs, Hungary

**Keywords:** dihydroxybenzenes, acetic acid, ethyl acetate, microelectrode, voltammetry

## Abstract

An electrochemical investigation of 1,2- and 1,4-dihydroxybenzenes was carried out with platinum macro- and microelectrodes using square wave and cyclic voltammetry techniques. Furthermore, the effect of the two solvents—acetic acid and ethyl acetate—was compared. When using square wave voltammetry, signals only appeared at lower frequencies and only when the supporting electrolyte was in excess, as expected due to the relatively low permittivity of the used solvents. The behavior of hydroquinone and catechol did not differ significantly from that of their derivatives (dihydroxybenzaldehydes, dihydroxybenzoic acids and 2′,5′-dihydroxyacetophenone). When the cyclic voltammetric experiments using a microelectrode were extended to higher anodic potentials, electrode fouling was very significant in ethyl acetate after the potential region where steady-state oxidation to the corresponding quinone occurs. The substituent effect was not significant here either, which was proven by using different functional groups in different positions. In contrast, the position had a dramatic influence on the susceptibility to electropolymerization, as 1,2-dihydroxybenzenes—independent of the nature of the substituent on the benzene ring—deactivated the electrode, while 1,4-dihydroxybenzenes did not, possibly due to the different solubilities of the polymers formed from the primary oxidation product (quinones). A user-friendly analytical procedure is also proposed that uses an electropolymerization reaction and does not require frequent cleaning of the electrode via polishing, which is required usually especially with a microelectrode.

## 1. Introduction

The use of derivatives of dihydroxybenzenes in many applications has been examined due to their favorable redox properties, especially when the two hydroxyl groups are in the 1,2 and 1,4 positions. The role of benzene ring substituents is highlighted by the solvation properties of the substrates and their electropolymers. **For example**, the presence of carboxyl groups in dihydroxybenzoic acids alters their pK_a_ values, which are predominantly between 2 and 3, allowing a high degree of dissociation and allowing for investigations in aqueous environments. Generally, 1,2- and 1,4-dihydroxybenzenes undergo a reversible two-electron oxidation–reduction reaction, followed by proton transfer. However, the proton number depends on the pH; thus, their reaction becomes pH-independent in strongly basic conditions, as shown for dihydroxybenzoic acids [1]. Furthermore, the quinone oxidation products can be oxidized further on glassy carbon electrodes to hydroxylated derivatives. As dihydroxybenzoic acids can dissolve in water in significant amounts, analytical methods have been developed for their quantification with modified electrodes [2,3,4,5,6,7]. The carboxy derivatives of dihydroxybenzenes can also serve as starting materials for electrosynthesis reactions; for example, those that can react with acetylacetone to facilitate the green synthesis of benzofuran derivatives [8].

In electropolymerization reactions, phenols play a predominant role. They have one or more hydroxyl groups on the benzene ring; these functional groups are oxidized in the first step. They are the most commonly used compounds to produce polymers with ether linkages between the benzene rings via electrooxidation. Therefore, extensive research has been conducted on phenols thanks to their many possible applications, like corrosion barrier deposits, size-exclusion polymers and electrode-modifying layers in the determination of compounds. Examples of dihydroxybenzenes containing two hydroxyl groups in the 1,3 positions relative to each other are resorcinol and its derivatives, and they are very susceptible to electrode fouling when they are electrochemically oxidized. In a recent work, we investigated the resorcinol derivatives 3,5-dihydroxybenzoic acid and 2’,6’-dihydroxyacetophenone in dimethyl sulfoxide. The acetophenone derivative formed only a deposit, showing the importance of the solvent in solvation of the oligomers formed on the electrodes [9]. The layer formed from the acetophenone derivative proved very useful in alleviating the current noise originating from stirring the solution, so reliable amperometric methods could be developed by utilizing it as a modifying layer.

Generally, when phenols undergo anodic oxidation, polymerization is initialized, and the presence of a phenol or resorcinol part is required within the monomer. In each case, the process can be influenced by not only the nature of the monomer and solvent but also the monomer concentration, solution pH and presence of additives. These parameters influence deposit growth on electrodes; therefore, these reactions must be optimized. By using cyclic voltammetry (changing the number of cycles) and potentiostatic methods (changing the electrolysis time), the thicknesses of coatings are controlled; this is another necessary factor for optimization. Natural antioxidants have been widely examined in polymer formation, and polymeric coatings of *p*-coumaric acid and naringin exhibit electroactive behavior [10,11]. The catechol fragment found in both molecules is responsible for this electrochemical activity, and the appearance of a pair of peaks reinforces the reversible redox behavior; the anodic and cathodic peaks have practically the same height in each scan. These deposits usually have electrocatalytic properties, as the quinone moieties in their oxidized form may act as redox mediators for numerous analytes.

The solubility of dihydroxybenzaldehydes is highly limited in the aqueous phase, and it also depends significantly on the solution pH. A basic solution allows for their utilization in water. As their electropolymers have very low solubility in aqueous environments, their excellent capability in redox-mediated processes has been utilized, especially in the case of 3,4-dihydroxybenzaldehyde. The latter compound was electrochemically polymerized on the surface of a glassy carbon electrode, and the modified electrodes showed analytical usefulness in the quantification of NADH [12,13,14], fructose [15], ascorbic acid [16], L-lactate [17], formate and glucose-6-phosphate [18], L-phenylalanine [19], NADH and ascorbate [20] and hydrazines [21,22]. The electron transfer between the electrode and solution due to covalently bound 2,5-dihydroxybenzaldehyde also enhances the development of a glucose-fuel-cell-containing bioanode, which is the modified electrode [23].

For decades, low-permittivity solvents have been studied due to reasons such as their solvation ability towards selected compounds. The investigated acetic acid has a relative permittivity of 6.2, and that of ethyl acetate is 6.0814 [24]. These two solvents readily dissolve hydrophobic compounds and their apolar matrix, if necessary, and they have a relatively wide potential window for oxidation reactions. Glacial acetic acid serves as an appropriate environment that could benefit from the favorable properties of acetate salts, as the acetate ion is a strong base in this condition, and its addition to acetic acid significantly improves analytical techniques. For example, in the work of Michalkiewicz et al., an acetate electrolyte served as an excellent choice in the determination of apocynin [25]. An increase in the molar fraction of acetic acid in acetic-acid–water mixtures leads to an increase in the oxidation potential of benzylamines, suggesting that the transition state is polar [26]. The permittivity can be increased by mixing acetic acid with a higher permittivity solvent; in this way, the preservation of favorable solvation properties for apolar compounds and parabens elevates their voltammetric determination in acetic-acid–acetonitrile mixtures [27]. Acetic acid as a solvent has also proven useful for the electroanalysis of α-tocopherol [28]. Ethyl acetate has limited miscibility with water, so in the work of Hardcastle et al., this solvent was utilized in solvent extraction for the detection of copper ions in aqueous samples with the aid of a complexing ligand [29]. Acetic acid has also illustrated its usefulness in the anodic reactions of numerous dissolved materials; it also exhibits electroactivity in the cathodic potential range, where its concentration can be determined by the **reduction of its** acidic proton. Through this cathodic reaction, its adsorption on activated carbon can be investigated [30].

In electroanalytical chemistry, the square wave voltammetry technique is widely used due to its relatively high sensitivity. The utilization of a square wave potential program has led to the development of powerful analytical procedures. In fact, square wave voltammetry has become a powerful technique to minimize the disturbance of the background current, in other words, to markedly reduce the signal-to-noise ratio. For example, in the work of Hooper et al., such a potential program was used for the time-dependent amperometric detection of selected analytes coupled with capillary electrophoresis [31]. This technique ensured a high degree of separation of the signal from the disturbance caused by the electric field attributable to the electrophoresis necessary for analyte separation.

A reduction in electrode size to the micrometer level provides numerous advantages for low-permittivity solvents. For decades, there has been a considerable interest in the application of microelectrodes for the examination of electrochemical reactions in such media, as well as in conditions where no supporting electrolyte is deliberately added. In the latter case, an “excess electrolyte zone” develops in front of the electrode from the presenting ionic impurities. The most important advantage of the use of microelectrodes in such conditions is the significant reduction in ohmic potential drop. The magnitude of this depends on the solution resistance and the expected current signal. In terms of the electrode shape, more types of micrometer-sized electrodes exist, for example, microdisc, cylindrical and microband electrodes. Electroanalytical procedures frequently use cylindrical microelectrodes, the diameters of which are in the micrometer range, with a length generally in the millimeter range. The reason for this is that the influence of imperfections on the interface between the sealing insulating material and the electrode is minimized. A carbon microfiber electrode was applied successfully in two studies by Pingarrón’s research group in the detection of vanillin [32] and the pesticide thiram in ethyl acetate [33]. Platinum microelectrodes provided a good option for the detection of tocopherols and α-tocopheryl acetate in acetic acid [34]. Furthermore, in the same solvent, the antioxidants tert-butylhydroquinone, tert-butyl-4-hydroxyanisole and butylated hydroxytoluene were successfully quantified using pulsed voltammetric techniques [35].

Previous studies focused mainly on the electrocatalytic properties of dihydroxybenzenes and on their analysis. In this work, a procedure is provided for estimating the composition of a solvent mixture by using the voltammograms of a redox active compound. These studies may give more insight into the microenvironment of dissolved molecules, and for this purpose, cyclic and square wave voltammetry techniques were selected to compare their performance. 

## 2. Materials and Methods

Throughout this study, HPLC-grade solvents were used, and the solute materials were analytical reagent grade. For the voltammetric experiments, tetrabutylammonium perchlorate (TBAP) was used as the supporting electrolyte. Platinum electrodes (1 mm diameter macroelectrodes and 25 μm microelectrodes made from Pt discs) served as the working electrodes, a platinum–iridium wire was the counter electrode and a silver wire was the reference electrode. The three electrodes were compressed in a voltammetric cell, which was always a glass vial, and they were connected to a Dropsens potentiostat (Oviedo, Spain).

Before each measurement, the surfaces of the working electrodes were polished with a polishing cloth coated with an aqueous suspension of alumina. After thorough washing with deionized water, they were ultrasonicated to remove the residual contaminants arising from polishing. Finally, the electrodes were dried with thorough washing with dry acetone to remove water traces to minimize the introduction of water into the studied solutions.

## 3. Results and Discussion

### 3.1. Square Wave Voltammetric Investigations of Dihydroxybenzenes in Acetic Acid and Ethyl Acetate

During square wave voltammetry, the peak heights are mainly influenced by the frequency. In this technique, two potential pulses are applied; the direction of the first one is according to the studied process (oxidation or reduction), and during this period, pre-electrolysis occurs with a width of the same time duration as the pulse. This is followed by a second pulse with the same width as the previous one, and the current is also sampled at the end of this pulse similarly to the pre-electrolysis period. The difference in the two current samplings is displayed on the screen and used for analysis; this is the cause of the high sensitivity. Consequently, raising the pulse heights as much as possible leads to better compound detection. If the frequency is changed, the pulse widths also change according to the expression *f* = 1/(2*t*_p_), where *t*_p_ relates to the pulse widths of the forward and reverse pulses. The frequency can be used to determine the scan rate *v* through the formula *v* = *fE*_i_, where *E*_i_ is the potential increment, which also determines the speed of scanning between the initial and final potentials. The peak currents depend on the pulse widths according to the following equation [36]:

$$\Delta i_{p}=\frac{nFAD^{1/2}c^{*}}{\pi^{1/2}t_{p}^{1/2}}\Delta \varphi$$

In this expression, *n* is the number of electrons transferred during the charge transfer process, *F* is the Faraday constant, *A* is the electrode surface area, *D* and c* are the diffusion coefficient and concentration of analyte, respectively, and *φ* is a dimensionless peak current parameter. The latter parameter can be found in the corresponding table, which can also be found, for example, on certain websites [37].

**Figure 1:**
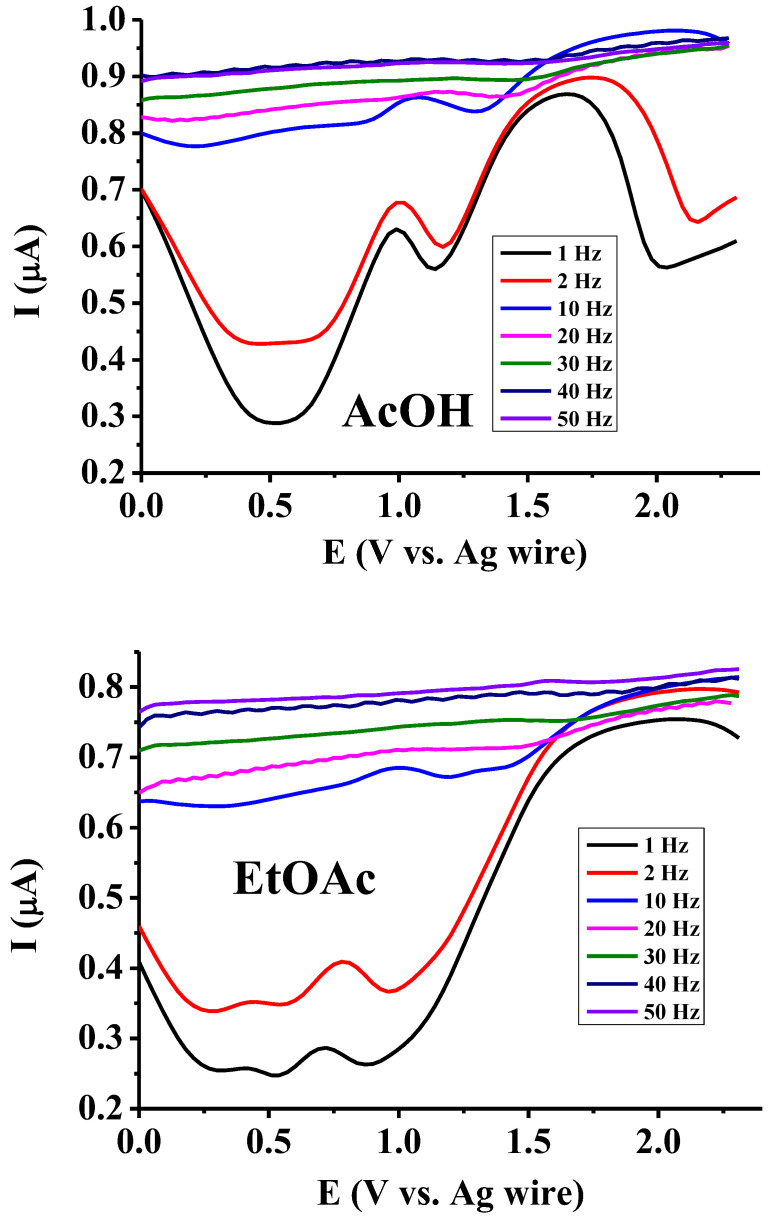
Square wave voltammetric curves in 1 mM solutions of 2,5-dihydroxybenzoic acid prepared with acetic acid and ethyl acetate by different frequencies by using 1 mm Pt macroelectrode (supporting electrolyte 50 mM TBAP, Estep = 0.03 V, E_pulse_ = 0.25 V).

The dependence of current signal heights on frequency is revealed in Figure 1. Evidently, only very low frequencies can provide useful curves for the characterization and analysis of compounds. At frequencies higher than 10 Hz, information will be lost. This phenomenon can be explained as follows: due to the low permittivity of the examined solvents, the solution resistance will be enhanced. Generally, to increase the sensitivity as much as possible, setting the height of the pulse potential *E*_pulse_ to the maximum value is advantageous due to the elevated diffusion flux to the electrode. Here, the opposite phenomenon is observed in the results. Thus, by applying a potential pulse at high frequencies, the widths of potential pulses will be shortened, leading to increased condenser currents compared to when very low frequencies are applied. On the other hand, due to the high solution resistance, using the exponential expression of the time constant e^−*t*/*RC*^, the whole expression takes a low value (*R* is the solution resistance and *C* is the electrode capacitance). As a result, as it is proportional to time, the condenser current drops slowly over time. In this technique, immediately after the application of the first pulse, a reverse pulse is applied, and taking the differences in their absolute values leads to practically the same condenser current. As these differences are large at higher frequencies, we can observe them develop into a flat curve well above zero current. To further support these observations, the oxidized product can be reduced quickly back to the analyte, but in these conditions, it is negligible. At low frequencies, the observed peaks at around 1.7 V and 2 V in acetic acid can be assigned to the oxidation of substrates; the small prepeaks are probably due to adsorbed species on the electrode. This indicates a notable difference between the two solvents, and differences in the permittivities are not reflected as much, indicating that other factors are also at play (see later).

**Figure 2:**
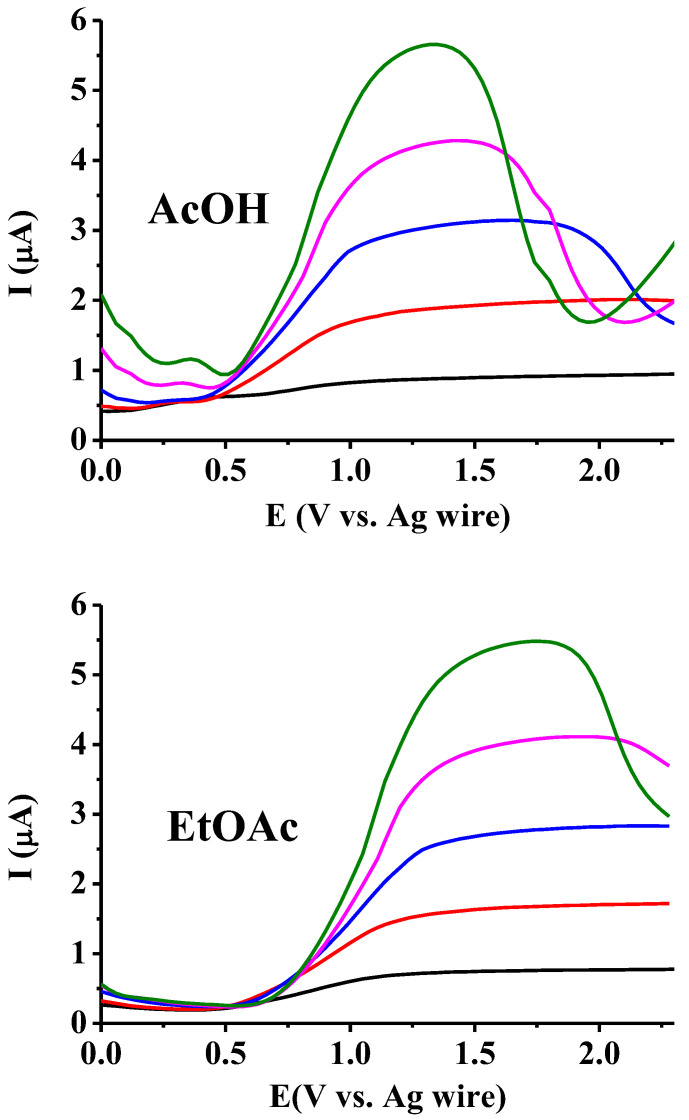
Dependence of the square wave voltammetric signal of 5 mM 2,5-dihydroxybenzoic acid by different TBAP concentrations in both studied low permittivity solvents (see in the figures), respectively (black: 10, red: 20, blue: 30, magenta: 40, green: 50 mM).

To further verify the above investigations, square wave measurements were carried out with the macroelectrode in both solvents by varying the concentration of the supporting electrolyte at a frequency of 1 Hz. The concentration of the redox active compound 2,5-dihydroxybenzoic acid was increased to 5 mM to increase the visibility of the voltammetric peak. As shown in Figure 2, the low supporting electrolyte concentration leads to flat curves, and the peak becomes increasingly sharp when its concentration is enhanced. The solution resistance decreases, and consequently the ohmic potential drops; thus, an increasingly higher pulse potential was imposed on the electrode. Meanwhile, the pulse heights enter the diffusion-controlled range, thus leading to sharper peaks. Basically, there is no significant difference between acetic acid and ethyl acetate; only the peak potentials shifted to more positive values in ethyl acetate by approximately 0.4 V (changing from about 1.4 V for acetic acid to about 1.8 V for ethyl acetate). This is possibly due to the reduced solvation ability of ethyl acetate molecules for the charged transition state (see [26]). The other difference is that the curves begin to peak only at a TBAP concentration of around 40 mM in ethyl acetate. This is an appropriate solvent for the associated ionic pairs of the supporting electrolyte, thus decreasing the degree of dissociation (there is only a subtle difference between the permittivities of the two solvents).

### 3.2. Microelectrode Studies of 1,2- and 1,4-Dihydroxybenzenes in Acetic Acid and Ethyl Acetate

**Figure 3:**
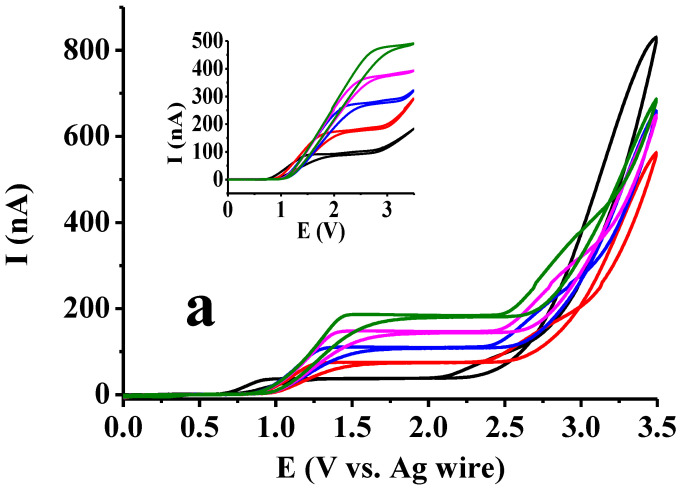

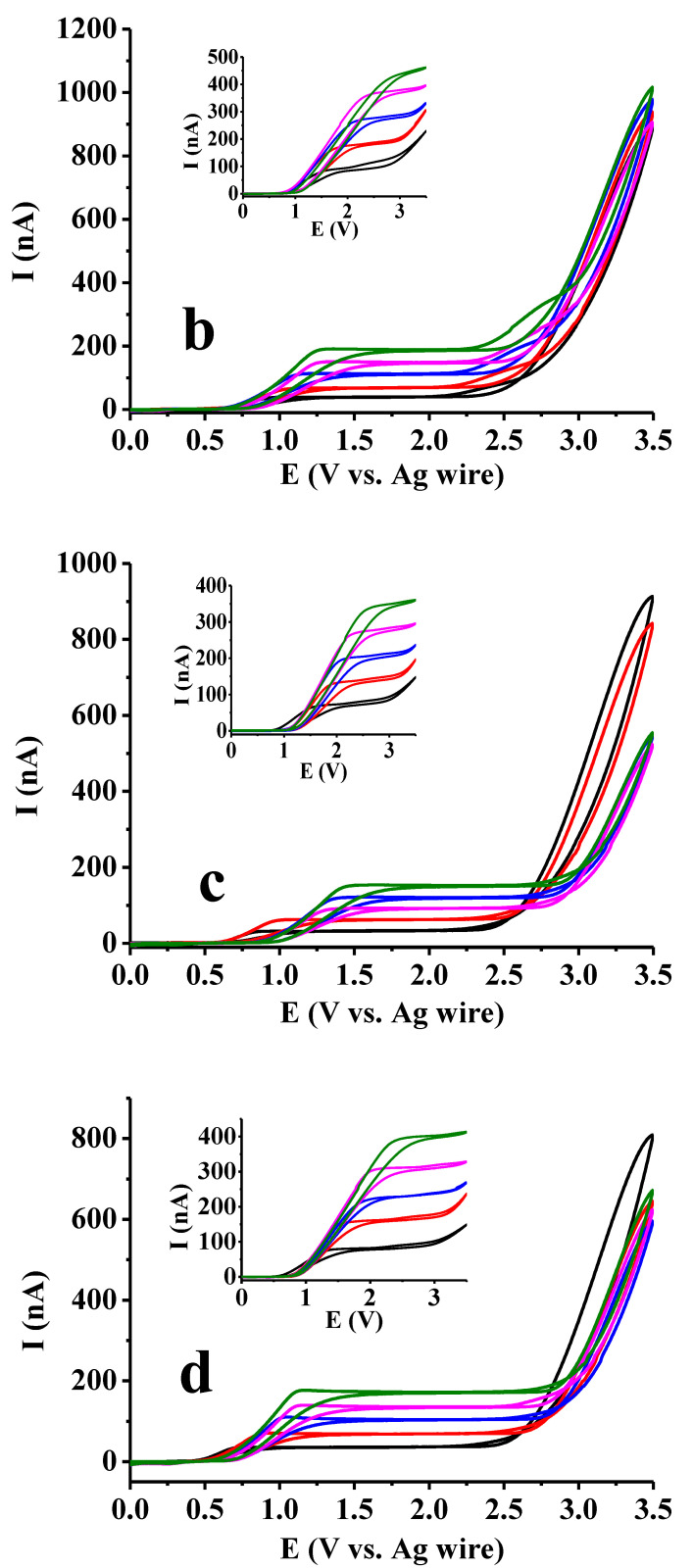

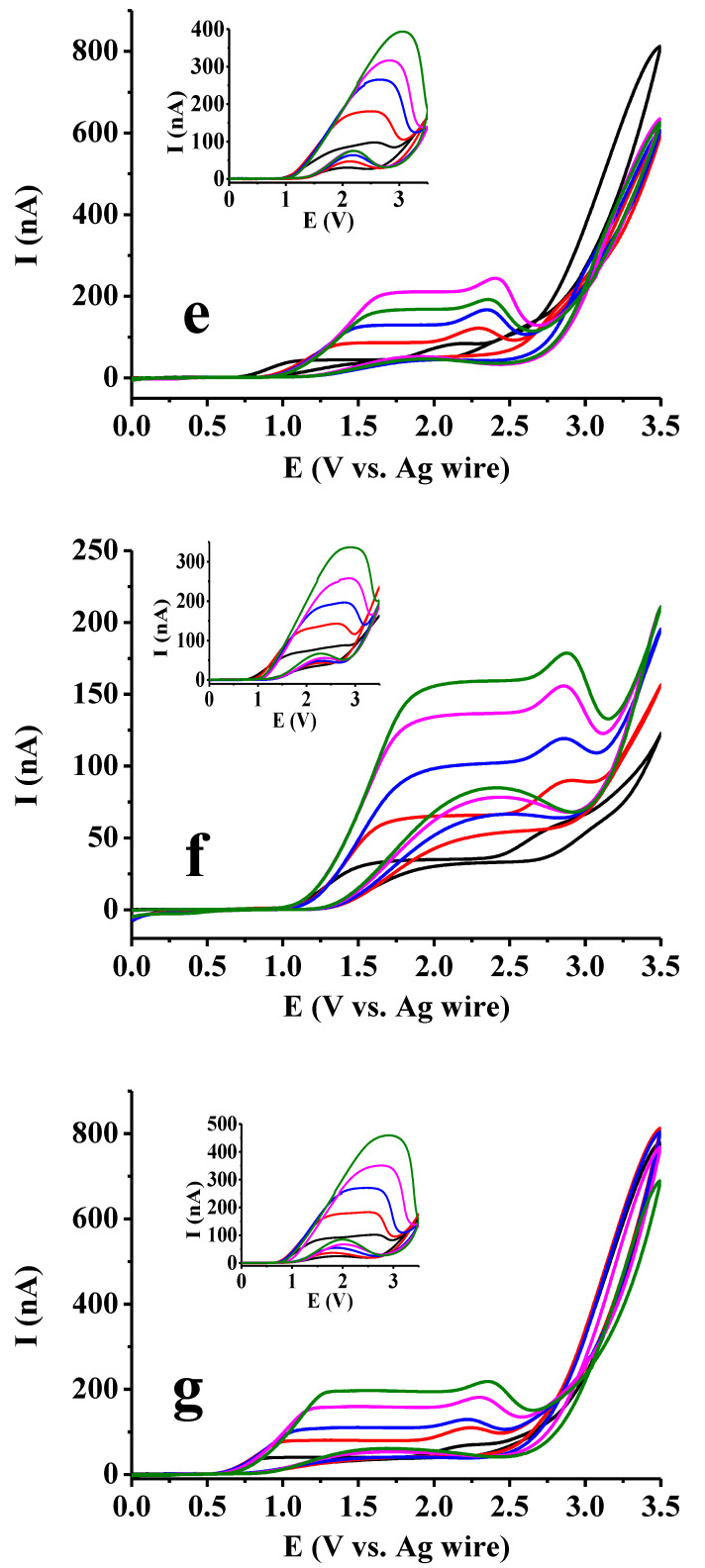
Effect of substituent and position of hydroxyl groups on the microelectrode voltammograms of 2,5-dihydroxyacetophenone (**a**), 2,5-dihydroxybenzaldehyde (**b**), 2,5-dihydroxybenzoic acid (**c**), hydroquinone (**d**), 2,3-dihydroxybenzaldehyde (**e**), 2,3-dihydroxybenzoic acid (**f**), catechol (**g**) in acetic acid (main graphs) and ethyl acetate (inset graphs). Black: 10 mM, red: 20 mM, blue: 30 mM, magenta: 40 mM, green: 50 mM substrate concentration.

It could be observed in the previous section that by using square wave voltammetry with a macroelectrode, there was no significant difference between the curves in acetic acid and ethyl acetate. Therefore, it would be very difficult to draw conclusions for solvent composition. Using square wave voltammetry always results in peaks in excess of the supporting electrolyte, independent of the electrode size, and of course, if the frequency is sufficiently low. Therefore, cyclic voltammetry was also applied. If there is no complication and the scan rate is not too high (generally lower than 200 mV/s), we obtain a sigmoidal curve. Its shape can be distorted if surface blocking occurs as a consequence of electrode reaction. A platinum microdisc electrode was used, as this type can be renewed by polishing if necessary. The microelectrode voltammetric curves are displayed in Figure 3 for more 1,2- and 1,4-dihydroxybenzenes by varying the substrate concentration between 5 and 25 mM incrementally with 5 mM by the steady 50 mM supporting electrolyte concentration and extending the potential window to 3.5 V with a 100 mV/s scan rate. The derivatives bearing formyl, carboxyl and acetyl groups and the bare substrates are compared. Basically, the figures show that the nature of substituents resulted in practically no differences. A visible difference could be observed in the shape of curves between dihydroxybenzenes, where the position of the two hydroxyl groups differed mainly in the ethyl acetate solvent. For the 1,4-dihydroxybenzenes, sigmoidal-shaped voltammograms could be observed in ethyl acetate, independent of the concentration. In contrast, for the 1,2-dihydroxybenzenes, well-developed peaks appeared in the same solvent. Additionally, the ohmic potential drops increased very significantly in ethyl acetate according to the substrate concentration; the plateaus were shifted very visibly to higher potentials where such signals appeared, noticeable when using hydroquinone and its derivatives.

However, in acetic acid, plateaus also appeared when studying 1,2-dihydroxybenzenes; small current drops appeared after them, indicating that a surface blocking process was initiated, but after that, the background current of the solvent could be seen with a sharp enhancement, as in the case of 1,4-dihydroxybenzenes. These findings are predominantly due to the solvent molecules undergoing the Kolbe reaction, leading to decarboxylation. Overall, the extension of the potential window enabled better differentiation between the two low-permittivity solvents. The quinone-producing oxidation reaction was responsible for the appearance of plateaus, and these quinones can be further oxidized to polymeric products. The formation of radicals leads to organic deposits and occurs simultaneously with the formation of acetyl radicals from acetic acid; thus, they can also terminate and therefore inhibit the deactivation of the electrode. Analyzing the shape of voltammograms, there was no sign of polymer formation from quinones of 1,4-dihydroxybenzenes. When hydroxyl groups were in the 1,2-position in the backward scan, visible small peaks appeared in ethyl acetate, suggesting that the rupture of deposits allowed the reactant to penetrate further, as their potentials are in the range of the diffusion-controlled process. These observations are in accordance with earlier results when using 3,4-dihydroxybenzaldehyde as a catechol derivative.

### 3.3. Effect of the Composition of Acetic Acid–Ethyl Acetate Mixtures on the Microelectrode Voltammograms of Catechol

In the previous section, it was established that the curvatures of microelectrode voltammograms of 1,2-dihydroxybenzenes were very different from those of 1,4-dihydroxybenzenes, especially in ethyl acetate. Therefore, the simplest 1,2-dihydroxybenzene catechol was chosen for further studies. Furthermore, the peak sharpness reached the highest value at a substrate concentration of 25 mM; therefore, this concentration was chosen as the steady value, alongside 50 mM of the supporting electrolyte.

The microelectrode should be cleaned before the first experiment, as described in the Materials and Methods section. After preparation of the stock solutions, the necessary volumes were pipetted into clean vials, and pure acetic acid and ethyl acetate were added to finally obtain the desired solvent composition in v/v%. Then, the voltammetric curves were recorded in each solution using the platinum microelectrode as the working electrode. The electrode was thoroughly washed with dry acetone after each experiment and allowed to dry. This latter step ensured that measurements in one solution did not influence the measurements in the next solution (see later). 

**Figure 4:**
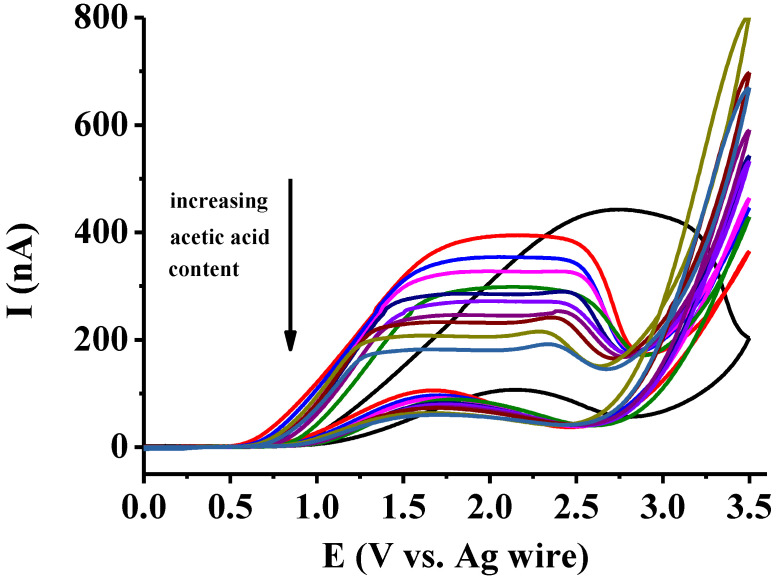
Dependence of the voltammograms of 25 mM catechol on the solvent composition (scan rate 100 mV/s, supporting electrolyte 50 mM TBAP, the curves regarding the content of ethyl acetate: black 100; red 90; blue 80; magenta 70; green 60; dark blue 50; violet 40; wine 30; brown 20; dark yellow 10; dark cyan 0 *v*/*v*%).

The voltammograms obtained for solutions in which the compositions of both solvents varied between 0 and 100 % are displayed in Figure 4. In accordance with the previous findings, anodic peaks appeared in solutions where the ethyl acetate content was high. The height and sharpness of these peaks decreased with increases in the acetic acid content. For all compositions, the curves of the backward scans were well below the ones in forward scans, indicating the electrode was blocked. Without deposit formation, the two parts of the curves should closely match each other on microelectrodes. Small peaks appeared in the backward scans due to the rupture of deposits, allowing the diffusion flux to the electrode to increase due to the change in tortuosity over time with the continuous solvation of blocking products. In compositions where the acetic acid content exceeded that of ethyl acetate, similar voltammograms could be measured with plateaus, as seen in previous studies on pure acetic acid. The shapes of the curves are useful for immediately determining whether the percentage of ethyl acetate or acetic acid is high. For acetic acid, in the concentration range between 50 and 100 *v*/*v*%, a dependence on the solution viscosity could easily be observed by a decrease in the plateau current heights. In some curves, at around 50 *v*/*v*% acetic contents, small peaks appeared after the plateaus, indicating some temporary blocking due to products.

It is important to emphasize the large difference in peak potential in ethyl acetate versus that of curves seen in acetic acid-containing mixtures. This suggests that the microenvironment of the protonated product of catechol, which forms immediately in the charge transfer reaction, and ethyl acetate molecules does not favor the dissolution of charged species. This process is significantly elevated by acetic acid molecules, and their presence highly contributes to the solvation of charged species, allowing the electrode to function more easily. This shift was previously observed for all derivatives of catechol in ethyl acetate, in contrast to acetic acid (see Figure 3).

**Figure 5:**
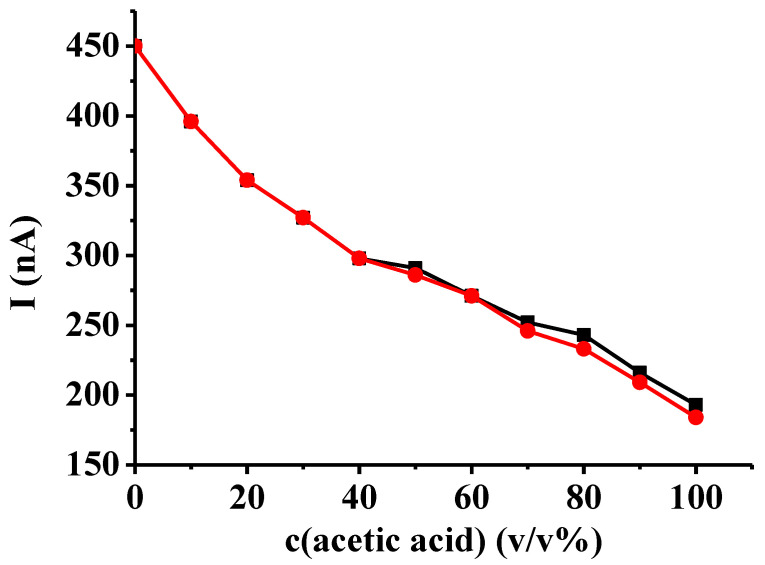
Calibration plots of solvent composition using the voltammetric signal of catechol (black: maximum current signal, red: maximum current signal and its heights when plateaus appeared).

Two types of calibration were studied to determine which is more sensitive to changes. One of the data series consisted of the maximum currents, while the other one included the plateau currents instead of the maximum currents where they appeared. The maximum currents were not those registered at 3.5 V, as they were poorly reproducible; they were instead the additional peak signals due to the temporal blocking seen in certain curves at around 2.4 V. As can be seen in Figure 5, the latter showed larger differences between the different compositions. This clearly shows that the electropolymerization reaction is highly sensitive towards the presence of acetic acid at lower percentages. The two curves closely resemble each other in this range, as only peaks appeared.

As sample addition requires the external addition of a redox active material and a supporting electrolyte, a solution was prepared by adding 2 ml of acetic acid and ethyl acetate to solid materials weighed in appropriate amounts with a micropipette, resulting in a 50 *v*/*v*% mixture of acetic acid and ethyl acetate. The redox active material catechol and supporting electrolyte were present at concentrations of 25 mM and 50 mM, respectively. The currents exhibited subtle differences to those for solutions containing dissolved materials, so the previously used calibration procedure was applied. Regarding the reproducibility of curves, very small deviations could be observed, so a slight adjustment was made to the electrode to completely restore the initial conditions in front of the microdisc. Here, we refer to a previous section, in which a procedure was described that allows for the rapid dissolution of blocking products. Electropolymerization only affects the curvature of voltammograms on the timescale of recording, allowing for easy observation of the phenomena, increasing the user-friendly nature of the protocol and eliminating the need for large equipment for techniques other than electrochemistry.

## 4. Conclusions

In this paper, we illustrated that the mostly undesired process of electrode deactivation caused by polymerization can be utilized in an analytical procedure. The differences in the solvation abilities of the two solvents with regard to products of a redox active molecule may prove useful for quantification. In other words, the appropriate choice of a redox active molecule with some degree of susceptibility to polymerization provides possibilities to estimate the composition of other mixtures as well. In summary, cyclic voltammetric studies highlight that, in many cases, the degree of deviation from the regular voltammograms helps estimate the solvent composition, especially when microelectrodes are used. In fact, electropolymerization reactions are highly appropriate for triggering such characteristic curvature of curves, as shown in this work.

## Data Availability

The data presented in this study are available on request from the corresponding author.
